# 'Correction:' Serum transforming growth factor beta-1 (TGF-beta-1) levels in diabetic patients are not associated with pre-existent coronary artery disease

**DOI:** 10.1186/1475-2840-6-19

**Published:** 2007-07-25

**Authors:** Beatriz D Schaan, Alexandre S Quadros, Rogério Sarmento-Leite, Giuseppe De Lucca, Alexandra Bender, Marcello Bertoluci

**Affiliations:** 1 Experimental Medicine Service, Institute of Cardiology of Rio Grande do Sul/University Foundation of Cardiology, Porto Alegre, Brazil; 2 Federal University of Rio Grande do Sul, Porto Alegre, Brazil; 3 Internal Medicine Unit, Hospital de Clínicas de Porto Alegre, Porto Alegre, Brazil; 4 Av. Princesa Isabel, 370 – Santana – Porto Alegre, Brazil

## Abstract

**Background:**

The association between TGF-β1 levels and long-term major adverse cardiovascular events (MACE) in patients with coronary artery disease (CAD) is controversial. No study specifically addressed patients with CAD and diabetes mellitus (DM). The association between TGF-β1 levels and long-term major adverse cardiovascular events (MACE) in patients with coronary artery disease (CAD) is controversial. No study specifically addressed patients with CAD and diabetes mellitus (DM).

**Methods:**

Patients (n = 135, 30–80 years) referred for coronary angiography were submitted to clinical and laboratory evaluation, and the coronary angiograms were evaluated by two operators blinded to clinical characteristics. CAD was defined as the presence of a 70% stenosis in one major coronary artery, and DM was characterized as a fasting glycemia > 126 mg/dl or known diabetics (personal history of diabetes or previous use of anti-hyperglycemic drugs or insulin). Based on these criteria, study patients were classified into four groups: no DM and no CAD (controls, C n = 61), DM without CAD (D n = 23), CAD without DM (C-CAD n = 28), and CAD with DM (D-CAD n = 23). Baseline differences between the 4 groups were evaluated by the χ^2 ^test for trend (categorical variables) and by ANOVA (continuous variables, post-hoc Tukey). Patients were then followed-up during two years for the occurrence of MACE (cardiac death, stroke, myocardial infarction or myocardial revascularization). The association of candidate variables with the occurrence of 2-year MACE was assessed by univariate analysis.

**Results:**

The mean age was 58.2 ± 0.9 years, and 51% were men. Patients with CAD had a higher mean age (p = 0.011) and a higher percentage were male (p = 0.040). There were no significant baseline differences between the 4 groups regarding hypertension, smoking status, blood pressure levels, lipid levels or inflammatory markers. TGF-β1 was similar between patients with or without CAD or DM (35.1 ×/÷ 1.3, 33.6 ×/÷ 1.6, 33.9 ×/÷ 1.4 and 31.8 ×/÷ 1.4 ng/ml in C, D, C-CAD and D-CAD, respectively, p = 0.547). In the 2-year follow-ip, independent predictors of 2-year MACE were age (p = 0.007), C-reactive protein (p = 0.048) and systolic blood pressure (p = 0.008), but not TGF-β1.

**Conclusion:**

Serum TGF-β1 was not associated with CAD or MACE occurrence in patients with or without DM.

## Background

Atherosclerosis is related to lipid abnormalities, platelet activation, thrombosis, endothelial dysfunction, inflammation, oxidative stress and altered matrix metabolism [1], among other disturbances. Several risk factors are known to play a role in this intricate cascade, and diabetes mellitus (DM) is associated with a 3-fold increase in the risk of death or myocardial infarction due to coronary artery disease (CAD) [2]. There is considerable debate as to whether the pathogenesis of atherosclerosis in diabetics is different, or whether DM simply accelerates this process. The identification of specific pathophysiologic features in diabetics could lead to a targeted approach to control disease progression in this set of patients.

A number of inflammatory cytokines mediate atherosclerosis, and TGF-β1 modulates important events like macrophage and fibroblast chemotaxis, suppression of lymphocyte function, collagen synthesis and stimulation of extracellular matrix synthesis [3-7]. Nikol et al demonstrated increased expression of TGF-β1 in human atherosclerotic plaques, and it was subsequently demonstrated that diabetic patients presenting with an acute myocardial infarction revealed decreased smooth muscle cells (SMCs), increased macrophages and TGF-β1 into the culprit lesion [8,9].

Other studies showed that reduced plasma levels of TGF-β1 in patients with angiographically proven CAD [10-12], and TGF-β1 levels were also found to be inversely related to the extend of coronary disease [13]. Tashiro et al observed that lower TGF-β1 levels were associated with increased risk of long-term major adverse cardiovascular events (MACE), suggesting that plasma levels of this citokyne may have prognostic significance in patients with CAD [14]. However, other studies failed to demonstrate an association between TGF-β1 and atherosclerotic disease [15], and no study was specifically addressed to diabetic patients. The aim of the present study is to evaluate the association between serum TGF-β1 and CAD in patients with DM, and its influence in long-term MACE incidence.

## Methods

### Subjects

Patients included were referred for coronary angiography by their attending physicians between December 2002 and December 2003. Inclusion criteria were age between 30 and 80 years, chest pain and documented myocardial ischemia in the non-invasive evaluation. The exclusion criteria were acute myocardial infarction in the last 60 days, previous heart transplantation or revascularization procedure, use of anticoagulants, known malignant neoplasia and haemodialysis. Each subject gave written informed consent and the study protocol was approved by the Hospital Ethics Committee.

On the day of the coronary angiography, a clinical questionnaire was applied and a physical examination was performed. A 12 h-fasting blood sample collection and a morning spot urine analysis were done before coronary angiography. Blood samples were collected in tubes without conservants, kept at room temperature for 3 hours and then stored at 4°C overnight. Serum aliquots were obtained after centrifugation (1000 G for 40 minutes at 4°C) and stored at -80°C until assay.

Based on the clinical history and on coronary artery disease severity, subjects were divided into four groups: no diabetes and no coronary artery disease (controls, C), diabetes and no coronary artery disease (D), coronary artery disease and no diabetes (C-CAD) and coronary artery disease and diabetes (D-CAD). Diabetes mellitus was defined as serum fasting glucose > 126 mg/dl or known diabetics, as evaluated by a personal history of diabetes or previous use of anti-hyperglycemic drugs or insulin.

Patients were followed-up either by clinical evaluation in an outpatient clinic, by interview with the attending physician or by telephone contact. The incidence of major adverse cardiovascular events (MACE) in the follow-up period was recorded. MACE was defined as the occurrence of cardiac death, ischemic stroke, myocardial infarction (AMI) or the need for a revascularization procedure (either percutaneous or surgical). Myocardial infarction was defined according to the criteria of the American College of Cardiology [16].

### Measurements of TGF-β1 levels

For the measurement of serum active TGF-β1 levels we used a solid phase ELISA designed to measure biologically active TGF-β1 (R&D Systems, Abingdon, UK). On the day of the assay, serum samples (0.5 ml) were acidified to a pH of 2–3 with 100 μl 1N HCL for 10 minutes to activate latent TGF-β, and then re-neutralized to pH 7–8 with 100 μl 1.2N NaOH/0.5 M HEPES. Results were expressed in ng/ml. The mean intra and inter assay coefficients of variation were respectively 2.0% and 13.1%.

### Biochemical analysis

Fasting blood samples were obtained for glucose, total cholesterol, high-density cholesterol, triglycerides, creatinine, and laboratory measurements were performed using automated enzymatic commercial kits (Roche, Manhein, GE). Fibrinogen was evaluated on a Fibrintimer II (Dade Behring Inc., Newark, Marburg, Germany) and processed in the auto-analyser (CA-540, Sysmex). Urinary albumin was evaluated in morning spot urine samples by immunoturbidimetry (antiserum to human albumin, Dade Behring Inc., Marburg, Germany) and the absolute concentration was considered. Levels higher than 17 mg/l were considered microalbuminuria. The concentration of low-density cholesterol (LDL-c) was calculated with Friedewald's formula. Serum insulin was determined by enzyme immunoassay commercial kits (Abbot-Murex, Park, IL, USA,), HbA1c by immunoturbidimetry (Roche, Manhein, GE) and ultrasensitive C-reactive protein (CRP) by nephelometry (nephelometer BN100, Dade Behring Inc., Marburg, Germany). Insulin resistance was assessed by the HOMA-IR (homeostasis model assessment of insulin resistance) in individuals who were not on anti-diabetic agents or insulin. The HOMA-IR is the product of the fasting plasma glucose (mmol/l) by plasma insulin (μ/ml), divided by a constant (22.5) [17].

### Coronary angiography

All coronary angiographies were performed at the Catheterization Laboratory of our institution according to standard routines. The coronary angiograms were evaluated by two experienced interventional cardiologists, unaware of the patients' glucose tolerance status. Angiographic analyses were performed in two orthogonal views with a validated digital quantitative coronary angiography system (Siemens Axiom Artis equipment; Munich, Germany). Significant coronary artery disease was considered to be present if an internal luminal narrowing greater than 70% was detected in at least one major coronary artery.

### Statistical analysis

Categorical variables were expressed as percentiles; continuous variables were expressed as the mean ± standard error of mean (SEM), unless otherwise specified. Differences between the four groups studied were evaluated by the χ^2 ^test for trend (categorical variables) and by ANOVA (continuous variables, post-hoc Tukey). TGF-β1 data were log-transformed before analysis. For all tests, a *P *value ≤ 0.05 was considered to be statistically significant. The association of candidate variables with the occurrence of 2-year MACE was assessed by univariate analysis, and those with a p ≥ 0.20 were included in a multiple regression logistic model.

## Results

The population of this study was comprised of 135 patients with a mean age of 58.2 ± 0.9 (30–80) years, of whom 51.1% were men. Patients with diabetes (D and D-CAD) had significantly more CAD than patients without diabetes (C and C-CAD; 50% vs 31.5%, p = 0.035). Table 1 shows baseline clinical characteristics of the studied subjects. Patients with CAD (D-CAD and C-CAD) had a higher mean age (p = 0.011) and a higher percentage were male (p = 0.040) than those without CAD. Body mass index was significantly higher in D *vs *the other groups. The other clinical characteristics assessed (hypertension and familial CAD history, percentage of patients who were smokers, statin/ASA/ACEI users, physically active and the mean systolic and diastolic blood pressure) were not statistically different between the four groups of patients. We observed a low overall use of statin in all groups, which could be related to the lack of an established previous diagnosis of CAD in many patients.

**Table 1:** Clinical characteristics of the studied patients

	C	D	C-CAD	D-CAD	P
*N*	61	23	28	23	
Mean age (years)	55.3 ± 1.4	58.2 ± 1.6	61.8 ± 2.0#	61.7 ± 2.0#	0.011
BMI (kg/m^2^)	28.07 ± 0.7	31.04 ± 1.0*	26.44 ± 0.8	27.27 ± 0.9	0.008
Male (%)	41.0	43.5	64.3 †	69.3 †	0.040
Hypertension history (%)	63.9	91.3	69.2	73.9	0.100
Familial CAD history (%)	68.3	63.6	59.3	69.6	0.831
Smoking (%)	31.7	30.4	23.1	26.1	0.857
Statin users (%)	18.0	8.7	25.0	17.4	0.512
AAS users (%)	63.3	82.6	71.4	78.3	0.284
ACEI users (%)	29.5	50.0	33.3	47.8	0.223
Physically active (%)	18.3	34.8	39.3	30.4	0.159
SBP (mmHg)	141.8 ± 4.7	134.4 ± 3.4	145.8 ± 7.3	140.2 ± 2.9	0.422
DBP (mmHg)	80.7 ± 2.5	84.3 ± 2.3	86.1 ± 4.5	87.0 ± 1.7	0.365

Data are means ± SEM or %

C: no diabetes and no coronary artery disease, D: diabetes and no coronary artery disease, C-CAD: coronary artery disease and no diabetes and D-CAD: coronary artery disease and diabetes; BMI: body mass index; ACEI: angiotensin-converting enzyme inhibitors; SBP: systolic blood pressure; DBP: diastolic blood pressure.

* P < 0.05 vs C-CAD; # P < 0.05 vs C; † P < 0.05 vs C and D

Table 2 shows baseline laboratory characteristics of the studied subjects. As expected, diabetics had higher fasting plasma glucose (p < 0.001), HbA1c (p < 0.001) and were more insulin resistant (higher HOMA-IR, p = 0.006). Lipids, fibrinogen, CRP and insulin levels were similar between the four groups studied.

**Table 2:** Laboratory characteristics of the studied groups

	C	D	C-CAD	D-CAD	P
*N*	61	23	28	23	
Plasma glucose (mg/dl)	93.1 ± 1.4	143.6 ± 14.5*	96.2 ± 2.2	141.8 ± 11.7*	<0.001
HbA1c (%)	5.71 ± 0.05	6.67 ± 0.35*	5.72 ± 0.06	7.08 ± 0.33*	<0.001
TC (mg/dl)	194.4 ± 5.5	201.7 ± 10.2	213.5 ± 7.9	219.6 ± 10.3	0.080
LDL-c (mg/dl)	111.9 ± 4.3	121.3 ± 8.3	126.2 ± 6.3	129.2 ± 8.2	0.144
HDL-c (mg/dl)	49.9 ± 1.5	46.6 ± 1.9	44.5 ± 2.2	45.4 ± 1.9	0.116
Non HDL-c (mg/dl)	142.7 ± 5.6	155.1 ± 9.3	168.4 ± 7.8#	174.2 ± 9.6#	0.009
TG (mg/dl)	160.9 ± 13.7	169.1 ± 15.8	216.4 ± 31.4	224.9 ± 30.7	0.082
Fibrinogen (mg/dl)	348.2 ± 11.4	335.7 ± 19.5	355.9 ± 17.4	353.2 ± 21.8	0.883
CRP (mg/l)	7.63 ± 1.27	4.48 ± 1.13	7.21 ± 2.61	8.57 ± 2.00	0.551
Insulin levels (μU/ml)	12.92 ± 1.3	21.25 ± 6.9	12.1 ± 1.4	17.6 ± 2.6	0.122
HOMA-IR	3.09 ± 0.34	9.06 ± 3.63*	2.99 ± 0.39	6.42 ± 1.16	0.006
TGF B (ng/ml)	35,1 ×/÷ 1,3	33,6 ×/÷ 1,6	33,9 ×/÷ 1,4	31,8 ×/÷ 1,4	0,547

Data are means ± SEM or n (%)

C: no diabetes and no coronary artery disease, D: diabetes and no coronary artery disease, C-CAD: coronary artery disease and no diabetes and D-CAD: coronary artery disease and diabetes; HbA1c: glycated haemoglobin; TC: total cholesterol; LDL-c: low-density lipoprotein cholesterol; HDL-c: high-density lipoprotein cholesterol; TG: triglycerides; HOMA-IR: model of homeostatic evaluation.

*P < 0.001 vs C and C-CAD; # P < 0.05 vs C

Figure 1 shows the baseline levels of serum TGF-β1. It is shown that they are similar between the studied groups, with or without angiographically proven coronary artery disease, diabetics or not.

**Figure 1:**
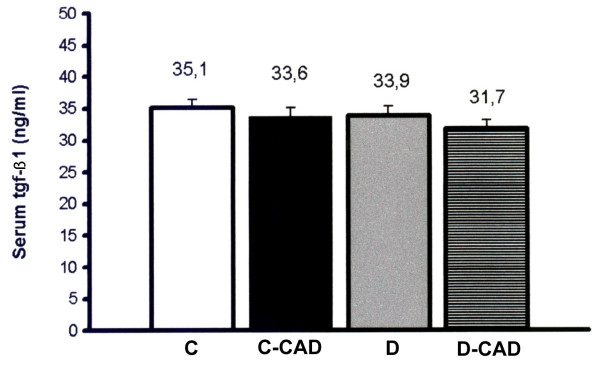
**Serum TGF-β1 in the four groups of patients studied**. C: no diabetes and no coronary artery disease (open bar), D: diabetes and no coronary artery disease (dark bar), C-CAD: coronary artery disease and no diabetes (gray bar) and D-CAD: coronary artery disease and diabetes (dashed bar). Data are expressed as geometric mean ×/÷ tolerance factor. p = 0.743 (ANOVA).

Clinical follow-up was completed in 123 patients (91.2%), and the mean follow-up period was 27.2 ± 6 months. From the possible associated variables evaluated, HbA1c, CRP, systolic blood pressure and angiographically proven CAD were significantly different in patients who developed MACE as compared with those who did not. These data are shown in Table 3. However, after multivariate analysis of candidate variables associated with 2-year MACE, those that were significantly associated with the endpoint studied were age (p = 0.007), CRP (p = 0.048) and systolic blood pressure (p = 0.008). Serum TGF-β1 was not significantly associated with MACE. These data are shown in table 4.

**Table 3:** Clinical, angiographic and laboratory characteristics of the patient population (n = 123) according to the occurrence of MACE in the 2-year follow-up

	MACE	No MACE	P
Age (years)	57.48 ± 1.5	60.77 ± 1.1	0.156
Male sex (%)	41.5	58.5	0.861
TGF-β1 (ng/ml)	33.82 ± 1.5	37.08 ± 1.5	0.14
HbA1c (%)	6.31 ± 0.2	5.91 ± 0.09	0.001
CRP (mg/dl)	9.14 ± 1.8	5.51 ± 0.8	<0.001
SBP (mmHg)	144.1 ± 3.4	136.7 ± 2.0	0.021
CAD %	28.9	71.1	0.025

SBP: systolic blood pressure; CAD: coronary artery disease

**Table 4:** Multivariate analysis of candidate variables associated with 2-year MACE

Variable	OR	CI 95%	Waldχ^2^	β	p
Age (years)	0.936	0.89–0.98	7.293	-0.066	0.007
TGF-β (ng/ml)	0.993	0.95–1.03	0.096	-0.007	0.756
HbA1c (%)	1.801	0.99–3.24	3.832	0.589	0.050
CRP (mg/dl)	1.059	1.00–1.12	3.927	0.057	0.048
SBP (mmHg)	1.035	1.00–1.06	7.006	0.034	0.008
CAD (presence or absence)	1.857	0.68–5.01	1.494	0.619	0.222

Constant			3.307	-5.230	0.069

CI: Confidence Interval; SBP: systolic blood pressure; CAD: coronary artery disease

## Discussion

The present study did not demonstrate a significant association between serum TGF-β1 levels and CAD in diabetic patients, or in controls. The independent predictors of 2-year MACE were increased age, C-reactive protein and systolic blood pressure, but not serum TGF-β1.

TGF-β1 biological activities include inhibition of cell proliferation, regulation of cell differentiation, stimulation of cell adhesion, extracellular matrix deposition [18,19] and inhibition of the growth and proliferation of vascular smooth cells [4], findings consistent with an anti-atherogenic role for TGF-β1. It also has a role in the regulation of the inflammatory process in the atherosclerotic plaque, being secreted by several cell types, including regulatory Th2 cells. It acts by antagonizing Th1 cell IF-gama production, an inflammatory response inductor [6]. Indeed, TGF-β1 plays a much more important role in the down-regulation of Th1 cell inflammatory response than in the atherosclerotic lesion size. Thus, large plaques do not necessarily mean a more advanced inflammatory process. According to that particular rationale TGF-β1 is a much more important inflammatory marker than an atherosclerotic marker, which agrees with the present data.

Several clinical studies have already demonstrated that patients with advanced atherosclerosis present lower plasma concentrations of TGF-β1 than those without CAD [11-14,20]. Conversely, Border and Ruoslahti showed that TGF-β1 could enhance atherogenesis by mediating excessive extracellular matrix accumulation [21] and by down-regulating thrombomodulin, promoting thrombogenesis at the sites of vessel wall injury, where it is released from platelets, smooth muscle cells and monocytes [22]. This is in accordance with clinical studies which showed high levels of active TGF-β1 levels in CAD [15]. However, none of the data mentioned above specifically addressed subjects with diabetes, which was the subject of the pesent work.

Although not classifying patients as diabetics or not, Wang et al demonstrated a positive correlation between active and total TGF-β1 levels and plasma glucose. *In vitro *studies showed that hyperglycemia induces TGF-β1 production in endothelial cells [23] and in mesangial cells of the kidney [24]. We suggest that TGF-β1 levels of our diabetic patients are raised in consequence of hyperglycemia, since their plasma glucose levels and HbA1c were higher than those of non-diabetics. Possibly in the diabetic population the hyperglycemia counteracts the atherosclerosis TGF-β1 reducing effect, masking the discriminatory role of this cytokine as found by other studies in non-diabetic subjects. Moreover, as aspirin medication correlates with an increase in active TGF-β1 concentration [11], the high percentage of subjects using the drug (71%) also could have masked its discriminatory role.

We could not stratify patients according to CAD severity, because of the small number of subjects in each studied group. It is possible that the relevance of TGF-β1 in discriminating CAD could only be observed in severe disease, as shown by other authors in CAD [13,25,26] and peripheral artery disease [27]. Using clinical criteria for the diagnosis of significant atherosclerotic vascular disease in hemodialysis patients, Stefoni et al showed reduced survival in a 24 month follow-up of patients with lower TGF-β1 levels at the beginning of the study [25]. Tashiro et al provided the unique evidence in literature that plasma concentrations of TGF-β1 have a prognostic significance in patients with angiographically-proven CAD, in a limited number of patients followed up for a mean period of 979 days [14]. This study did not show plasma TGF-β1 measurements in the patients' follow-up, but these values are supposed to be stable, according to the authors. Although the same drawbacks could be applied to the present study, we showed a different result, and therefore we question the significance of those findings.

A potential limitation of the present study could be the stability of TGF-β1 in peripheral blood. In a recent work, we showed that plasma TGF-β1 is quite stable in patients with diabetes and nephropathy, no changes being detected by strict 12-week blood pressure control despite a significant fall in urinary TGF-β1 levels [28]. Although it is possible that minor changes in TGF-β1 production from endothelial cells could not be detectable in serum where platelet TGF-β1 is abundant, greater decreases, as expected, are unlikely. Other possible limitation of the present study was the number of subjects studied, that did not permit to stratify the sample by age, searching for possible age-related physiological decrease in TGF-β1 levels described by other authors [29].

## Conclusion

In conclusion, serum TGF-β1 was not associated with CAD or MACE occurrence in diabetic patients. The role of TGF-β1 in atherosclerosis associated with diabetes needs further evaluation by means of longer prospective studies with a large number of patients.

## Competing interests

The author(s) declare that they have no competing interests.

## Authors' contributions

All authors have equally contributed to the conception and drafting of the manuscript.
